# Multimodal MRI-Based Triage for Acute Stroke Therapy: Challenges and Progress

**DOI:** 10.3389/fneur.2018.00586

**Published:** 2018-07-24

**Authors:** Oh Young Bang, Jong-Won Chung, Jeong Pyo Son, Wi-Sun Ryu, Dong-Eog Kim, Woo-Keun Seo, Gyeong-Moon Kim, Yoon-Chul Kim

**Affiliations:** ^1^Department of Neurology, Samsung Medical Center, Sungkyunkwan University School of Medicine, Seoul, South Korea; ^2^Department of Health Sciences and Technology, Samsung Advanced Institute for Health Sciences and Technology, Sungkyunkwan University, Seoul, South Korea; ^3^Stroke Center and Korean Brain MRI Data Center, Dongguk University Ilsan Hospital, Goyang, South Korea; ^4^Samsung Medical Center, Clinical Research Institute, Seoul, South Korea

**Keywords:** stroke, MRI, endovascular treatment, machine learning, triage

## Abstract

Revascularization therapies have been established as the treatment mainstay for acute ischemic stroke. However, a substantial number of patients are either ineligible for revascularization therapy, or the treatment fails or is futile. At present, non-contrast computed tomography is the first-line neuroimaging modality for patients with acute stroke. The use of magnetic resonance imaging (MRI) to predict the response to early revascularization therapy and to identify patients for delayed treatment is desirable. MRI could provide information on stroke pathophysiologies, including the ischemic core, perfusion, collaterals, clot, and blood–brain barrier status. During the past 20 years, there have been significant advances in neuroimaging as well as in revascularization strategies for treating patients with acute ischemic stroke. In this review, we discuss the role of MRI and post-processing, including machine-learning techniques, and recent advances in MRI-based triage for revascularization therapies in acute ischemic stroke.

## Introduction

Revascularization therapies, including rt-PA and EVT, have been established as the mainstay of treatment for acute ischemic stroke. It has become clear that consideration of heterogeneity among stroke patients is of importance in these therapies. Neuroimaging has been used as a triage tool for revascularization therapy in patients with acute stroke. The use of magnetic resonance imaging (MRI) for predicting the response to early revascularization therapy and for identifying patients in whom delayed treatment is appropriate is desirable.

During the past 20 years, there have been significant advances in both neuroimaging as well as in revascularization strategies for treating patients with acute ischemic stroke. In this review, the role of MRI discussed and the recent advances in MRI-based triage for revascularization therapies and in post-processing, including machine learning techniques, in these patients.

## Neuroimaging studies in the acute stroke intervention field: results from randomized controlled trials

**Figure 1:**
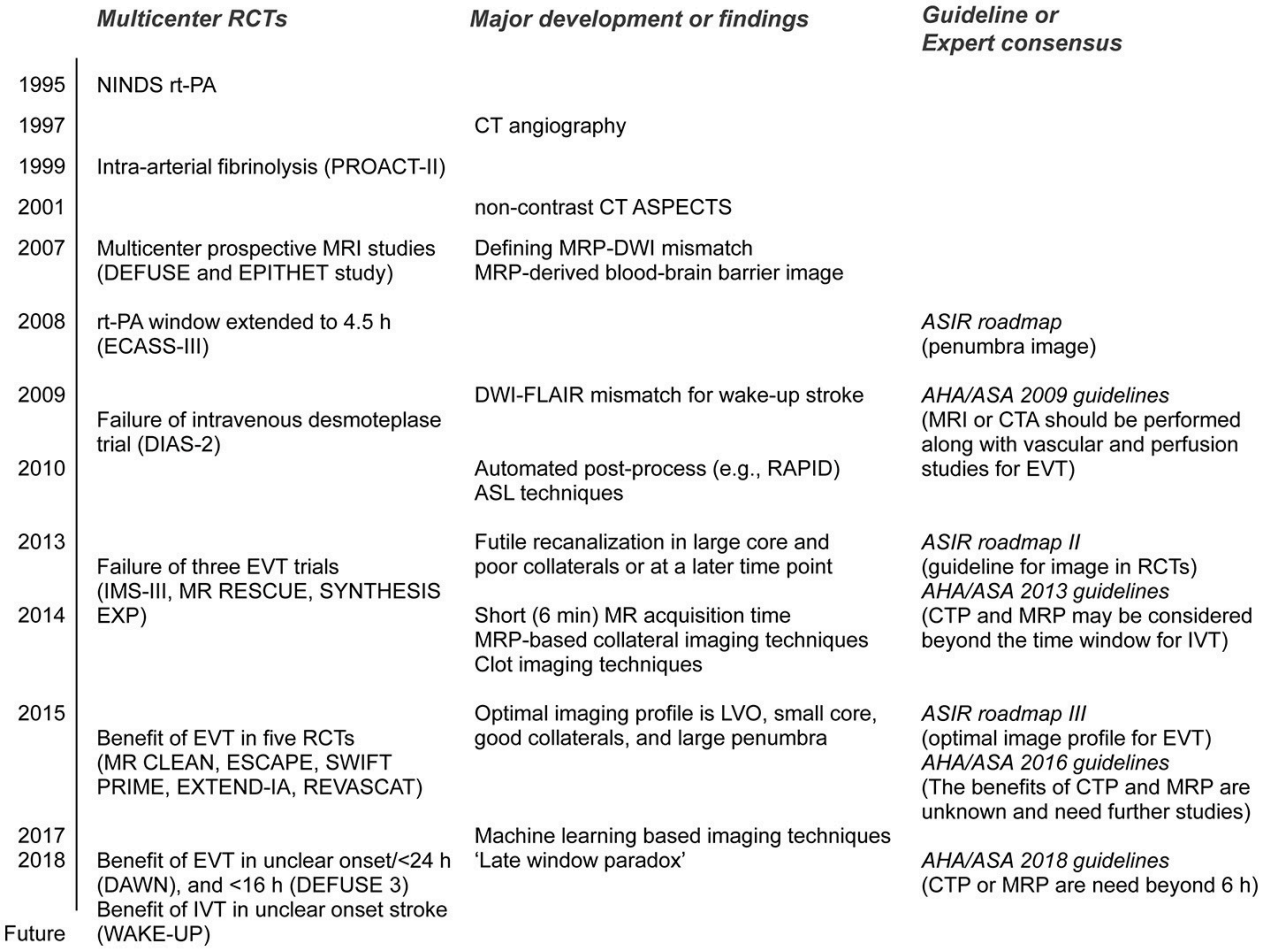
Major randomized controlled trials (RCTs) and corresponding guidelines in the field of acute stroke therapy. rt-PA, recombinant tissue plasminogen activator; ASPECTS, Alberta Stroke Program Early CT Score; MRP, magnetic resonance perfusion; DWI, diffusion-weighted image; FLAIR, fluid attenuation inversion recovery; ASIR, Acute Stroke Imaging Research; AHA/ASA, American Heart Association/American Stroke Association; DIAS-2, Desmoteplase in acute ischemic stroke-2; RAPID, RApid postprocess for PerfusIon and Diffusion; ASL, arterial spin labeling; LVO, large vessel occlusion; EVT, endovascular therapy. Please see Glossary for other abbreviations.

Previous intravenous rt-PA trials [the NINDS rt-PA (1) and ECASS-III (2) trials] have used non-contrast computed tomography (NCCT) images. Randomized controlled trials (RCTs) of EVT have implemented MRI or computed tomography perfusion/angiography (CTP/CTA) techniques, in addition to the NCCT Alberta Stroke Program Early CT Score (ASPECTS). However, the results of RCTs and the rapid evolution of neuroimaging techniques have led to significant changes in the international guidelines for neuroimaging in acute ischemic stroke over time (Figure 1).

Multicenter prospective MRI studies, including the DEFUSE and EPITHET trials for intravenous rt-PA given more than 3 h post-stroke, reported a significant association between recanalization and reduced infarct growth in patients with an MR perfusion–diffusion mismatch (3, 4). Expert consensus on the Acute Stroke Imaging Research (ASIR) Roadmap reported on the methodological issues in perfusion and penumbral imaging (5). The 2009 American Heart Association/American Stroke Association (AHA/ASA) guidelines recommended that MRI or CTA should be performed in conjunction with vascular and perfusion studies for EVT (6).

The IMS III (7), MR RESCUE (8), and SYNTHESIS expansion (9) trials were multicenter, prospective RCTs that failed to show a benefit from EVT for acute ischemic stroke. The potential reasons for this failure include time delays to angiographic reperfusion and the inclusion of patients with large core or no large vessel occlusion. In addition, the MR RESCUE trial used an algorithm that implements multiple diffusion-weighted imaging (DWI) and MR perfusion (MRP) parameters, but failed to show that patients with a favorable penumbral pattern on neuroimaging benefitted from EVT (8). Given the lessons from these aforementioned three RCTs, published 2013, the ASIR Roadmap II provided guidelines for the use of imaging in stroke clinical trials (10). In the AHA/ASA 2013 guidelines, the recommendations were changed to “CTP and MRP may be considered” (11).

Further phase III RCTs were conducted in 2015; these included the MR CLEAN (12), ESCAPE (13), EXTEND-IA (14), SWIFT PRIME (15) and REVASCAT (16) trials. The findings of these RCTs demonstrated overwhelming evidence of the benefit of EVT for treatment of acute ischemic stroke with a small core (as measured by the ASPECTS) and large vessel occlusion. The ASIR Roadmap III proposed the optimal imaging profile for EVT, based on the results of these recent positive RCTs: the presence of large vessel occlusion, a smaller core, good collaterals, and a large penumbra (17). After the success of the ASPECTS-based RCTs of EVT in 2015, the recommendations were again changed to “the benefits of CTP and MRP are unknown and need further studies” (18). For patients eligible for EVT, the 2016 AHA/ASA guidelines require the absence of bleeding and an ASPECTS of 6 points or more in NCCT, as well as the presence of causative occlusion of the internal carotid artery or proximal middle cerebral artery (18).

Very recently, the results of the phase III RCTs of EVT in an extended time-window showed a significant and remarkable functional recovery with EVT vs. that with medical treatment in carefully selected patients (19, 20). EVT was initiated between 6 and 16 h after onset in patients with a target mismatch in the DEFUSE 3 trial (20), and 6–24 h after onset in patients with mismatch between clinical presentation and DWI/CTP in the DAWN trial (19). In these trials, the benefits of EVT persisted [or even increased, i.e., “late-window paradox” (21)] across the period when patients had a small core and large salvageable tissues. Based on these trials, the new 2018 guidelines recommended that CTP or DWI/MRP scans be obtained if the patient presents more than 6 h after his/her last known normal status and has large vessel occlusion (LVO), and to perform EVT when eligibility criteria from these trials were met (22).

## Implications of MRI-based triage on the number of patients receiving EVT

The beneficial effect of EVT has been confirmed in selected patients with acute ischemic stroke. However, a substantial proportion of patients are EVT ineligible (only 7–13% of acute ischemic stroke patients are eligible for EVT) (23), have failure of reperfusion (TICI 0–2a in 14–41% in five recent phase III RCTs) (12–16), or have futile reperfusion (26–49% showed a poor outcome, despite successful recanalization) (24). These findings indicate that imaging-guided tailored treatment may be beneficial in acute ischemic stroke. Although there have been significant advances in CT techniques, the advantages of MRI techniques make MRI more desirable for use (25, 26). The advantages and limitations of CT-based triage for EVT as well as the recent advances in MRI techniques are summarized in Table 1.

**Table 1:** Requirements for neuroimaging for EVT and recent advances in MRI techniques.

		**Imaging triages**	
**Requirements**	**Non-contrast CT**	**Advanced CT (CTA, CTP)**	**Advances in MRI**
**IS IT POSSIBLE?**			
Fast	Short scan time No need for post-processing	10–15 min for complete protocol	Short (6 min) acquisition time Automatic fast post-processing
Available	Widely available 24-7-365	Generally greater availability	Available in all comprehensive stroke centers
Reliable			
Standardization	Good	Parameters are being defined	Surrogate markers and their threshold and sequence are being defined
Inter-rate reliability	Modest (especially in the hyperacute phase)	Good	Machine-learning is being applied
**IS IT NECESSARY?**			
Stroke pathophysiology	ASPECTS for determining the extent of the core Dense MCA sign	CTP for core and penumbra CTA for collateral CTP for BBB damage	Perfusion pattern (Expected salvageable tissue) Collateral status (Predict response to treatment) BBB damage (Avoid hemorrhagic complications) Clot to treat (Need for adjuvant therapy)
Save more (proportion of extra cases) Unclear onset (1 in 3 patients) Delayed EVT (1 in 4 patients) ASPECTS <6 (1 in 8 patients)	Not applicable	Guided by not applicable CTA collaterals CTP, CTA ASPECTS	Guided by rFLAIR, Collateral, MRP Collateral, MRP DWI, Collateral, MRP
**IS IT TESTED?**			
Confirmed in RCTs	Role of ASPECTS confirmed	CTP (mismatch)-based EVT confirmed	MRI (mismatch)-based EVT confirmed

*CTA, computed tomography angiography; CTP, computed tomography perfusion; MCA, middle cerebral artery; BBB, blood–brain barrier; RCT, randomized controlled trial. Please see Glossary for other abbreviations*.

In EVT-eligible patients, MRI-based triage may increase the efficacy of EVT, at the expense of decreasing the number of patients receiving EVT by excluding patients with large lesions on DWI (27) (DWI is superior to any CT techniques in imaging the infarct core) (25). In contrast, MRI-based triage can also increase EVT use in patients considered ineligible under the current guidelines, as follows.

First, wake-up stroke occurs in one-fifth of patients with stroke; it was estimated that 58,000 patients with wake-up strokes presented to an emergency department in the U.S. in 2005 (3 million wake-up stroke cases worldwide) (28). In these cases, less time might have elapsed from the onset of stroke because circadian variation for stroke is well-known, with most cerebrovascular events known to occur during the morning (29). When also considering cases with an unknown time of symptom onset, for example, non-witnessed stroke with aphasia or disturbance of consciousness, it appears that the time of symptom onset is unknown in 14–35% of patients with acute stroke (28, 30–32). It is possible that some of these patients could benefit from revascularization therapies. A population-based study has shown that more than one-third of wake-up strokes would have been eligible for thrombolysis if arrival time were not a factor (28). MRI features combining fluid attenuation inversion recovery (FLAIR) sequences with DWI have been investigated as a surrogate marker for lesion age and a DWI-positive/FLAIR-negative mismatch pattern was identified in patients within 4.5 h of stroke onset in the middle cerebral artery territory, with high predictive values (33, 34). A recent RCT (WAKE-UP) showed that in patients with acute ischemic stroke with an unknown time of onset, intravenous rt-PA guided by a mismatch between DWI and FLAIR in the region of ischemia resulted in a significantly better functional outcome than the control group (35).

Second, patients could receive EVT if they have ASPECTS of ≥6 on NCCT and LVO on CTA, and if treatment can be initiated within 6 h of symptom onset (18). However, a significant proportion of patients arrived late at a comprehensive stroke center, where EVT can be performed, and some patients may require a longer procedural time. Data from US academic medical centers have shown that one-third of patients arrived more than 6 h after symptom onset (36). The ESCAPE trial showed that EVT improved functional outcomes and reduced mortality in patients with a small infarct core and moderate-to-good collateral circulation, up to 12 h after symptom onset (13). The HERMES investigators performed a meta-analysis of individual patient data from five recent RCTs of EVT, to test whether EVT is efficacious across a diverse population (i.e., a lower ASPECTS or longer onset-to-groin puncture time, etc.) (37). Patients with small cores (high ASPECTS) had a slower decline in benefit with longer symptom onset-to-reperfusion, than patients with larger infarct cores (38). In addition, reperfusion is related to a positive clinical outcome only if adequate collateralization can prevent infarction until the vessel can be recanalized. A good collateral status could thus feasibly extend the time-window for EVT (39–41). Patients with good collaterals as assessed by MRI showed a favorable outcome in terms of infarct growth at day 7 and modified Rankin score at day 90 (42, 43). Therefore, inclusion of patients with good collaterals, but not in those with larger cores, the time-window for EVT may be extended. Indeed, the results of the DAWN and DEFUSE 3 trials have extended the time-window in these patients to 16–24 h (19, 20).

Lastly, EVT is not recommended in patients with ASPECTS of <6 points on NCCT, according to the current guidelines (18). Data from the ECASS II study, in which 800 patients were randomized to rt-PA or placebo within 6 h of symptom onset, showed that the median ASPECTS value was 9, and that about one in seven patients showed ASPECTS of <6 on NCCT (44). Interestingly, the effect of rt-PA on functional outcome was not influenced by baseline ASPECTS, although patients with low ASPECTS have a substantially increased risk of thrombolysis-related parenchymal hemorrhage (44). In the DEFUSE 3 trial, there was no difference in the effect of EVT according to the ASPECTS score (<8 vs. ≥8) (20), which suggests that advanced image-guided selection could be considered in patients who have a low ASPECTS.

## Advances in MRI techniques for image processing and individual stroke pathophysiology

There have been significant advances in MRI techniques in terms of availability, acquisition (scanning and post-processing) time, direct visualization of cardinal features (the 4 Cs; i.e., [tissue]-clock, clot, collaterals, and core), and machine learning-based algorithm implementation.

### Availability

An NCCT scan is usually one of the first tests done in the evaluation of acute stroke. MRI takes longer and is often not available under emergency conditions, while NCCT has advantages in terms of fast acquisition time, widespread availability, and ease of interpretation in an emergency setting. However, MRI is available in all comprehensive stroke centers, where EVT can be performed. One single-center study showed that MRI-based triage for EVT is feasible in terms of the scan-to-groin puncture time, with acceptable rates of poor outcome and symptomatic hemorrhage (45). A recent randomized trial (General or Local Anesthesia in Intra Arterial Therapy, GOLIATH) showed that MRI selection for endovascular therapy can be accomplished rapidly and within a similar time frame as computed tomography-based selection (46). The door-to-MRI time can be reduced by a quality improvement process (47). Moreover, although a comprehensive MRI protocol can be implemented in ~20 min, a fast MRI protocol can be implemented in about 6 min, rivaling the time of any comprehensive acute stroke CT protocol (48). This fast MRI protocol includes DWI, FLAIR, gradient echo, and MR angiography, and MRP. The CTP image requires additional imaging time (2–3 min) and post-processing time (5–15 min) (49).

For clinical use, automated software that allows fast post-processing is mandatory, and is increasingly being used in clinical trials. For example, the RApid postprocess for PerfusIon and Diffusion (RAPID; Rapid Software Corporation, Grapevine, TX, USA), an automated software package for performing quantitative evaluation of the apparent diffusion coefficient to estimate the ischemic core and an MRP threshold of *T*_max_ > 6 s for defining critical hypoperfusion (50), has been used in several RCTs. Similarly, automated software for collateral assessment, the Fast Analysis SysTem for COLLaterals (FAST-COLL) (42), has been developed; it requires <5 min, and allows a clinical decision to be made at the workstation or bedside, based on the collateral grade.

### Visualization of individual stroke pathophysiology

**Figure 2:**
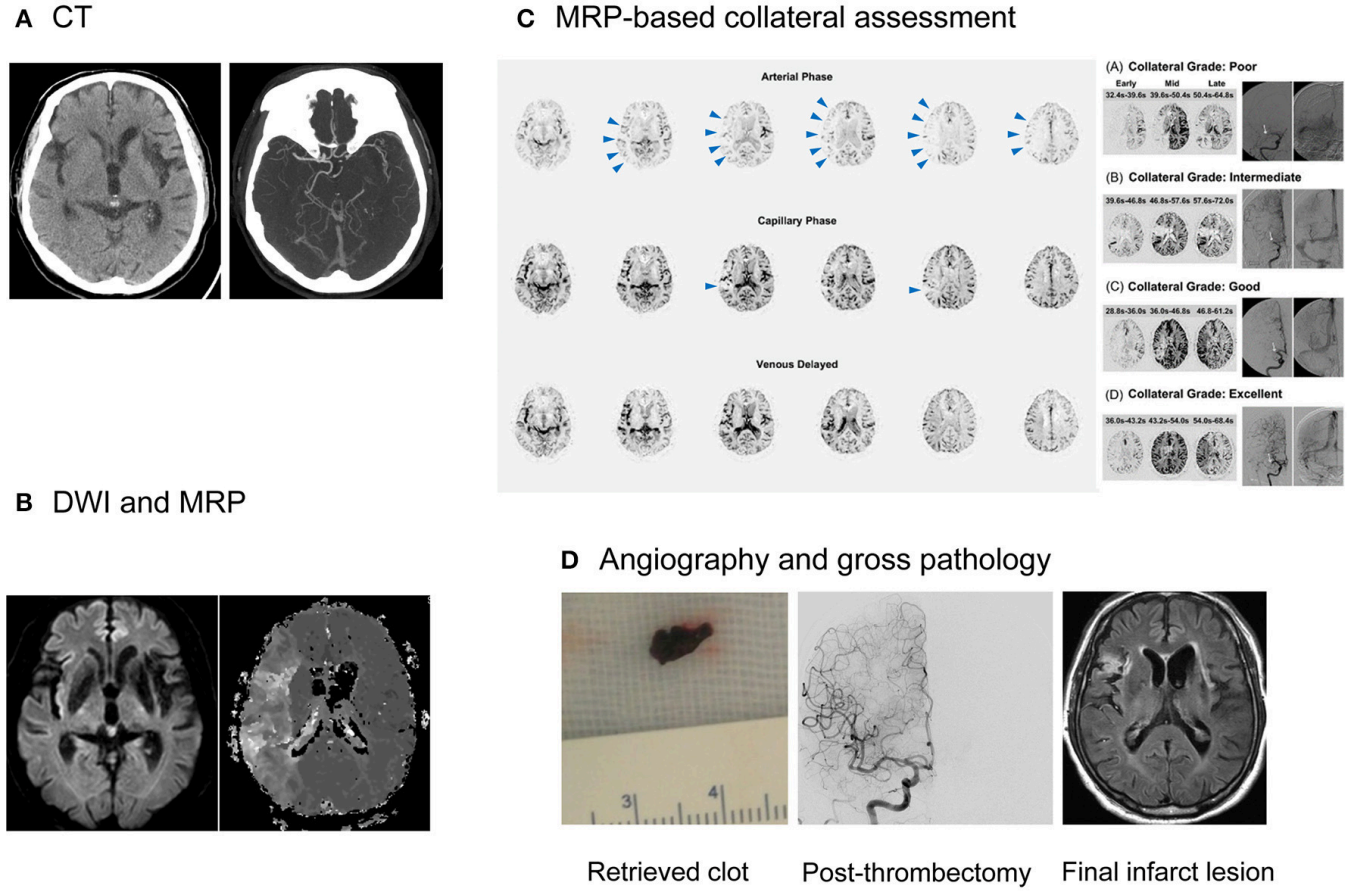
Collateral imaging-based delayed endovascular treatment. A 79-year-old female, with history of left MCA infarction and atrial fibrillation, presented with left-sided weakness and neglect symptoms at 940 min after symptom onset. **(A)** CT imaging documented right MCA occlusion, with an ASPECTS of 8. **(B)** MRI showed a significant mismatch, with a small core and large hypoperfusion regions. **(C)** MRP-based collateral imaging showed an excellent collateral flow, i.e., complete and rapid collateral flow to the vascular bed in the occluded MCA territory. Contrast staining in the lesional hemisphere was absent in the arterial phase (arrowheads), but evident in the capillary and venous phase. **(D)** Successful recanalization (mTICI grade 2b) was achieved and red clots were retrieved at the first passage of a stentriever at 1,090 min after the onset of symptoms. She was functionally independent and her modified Rankin score at the 90th day was 1. MCA, middle cerebral artery; CT, computed tomography; ASPECT, Alberta stroke program early CT Score; MRI, magnetic resonance imaging; DWI, diffusion-weighted imaging; MRP, MR perfusion; mTICI, modified treatment in cerebral ischemia.

**Figure 3:**
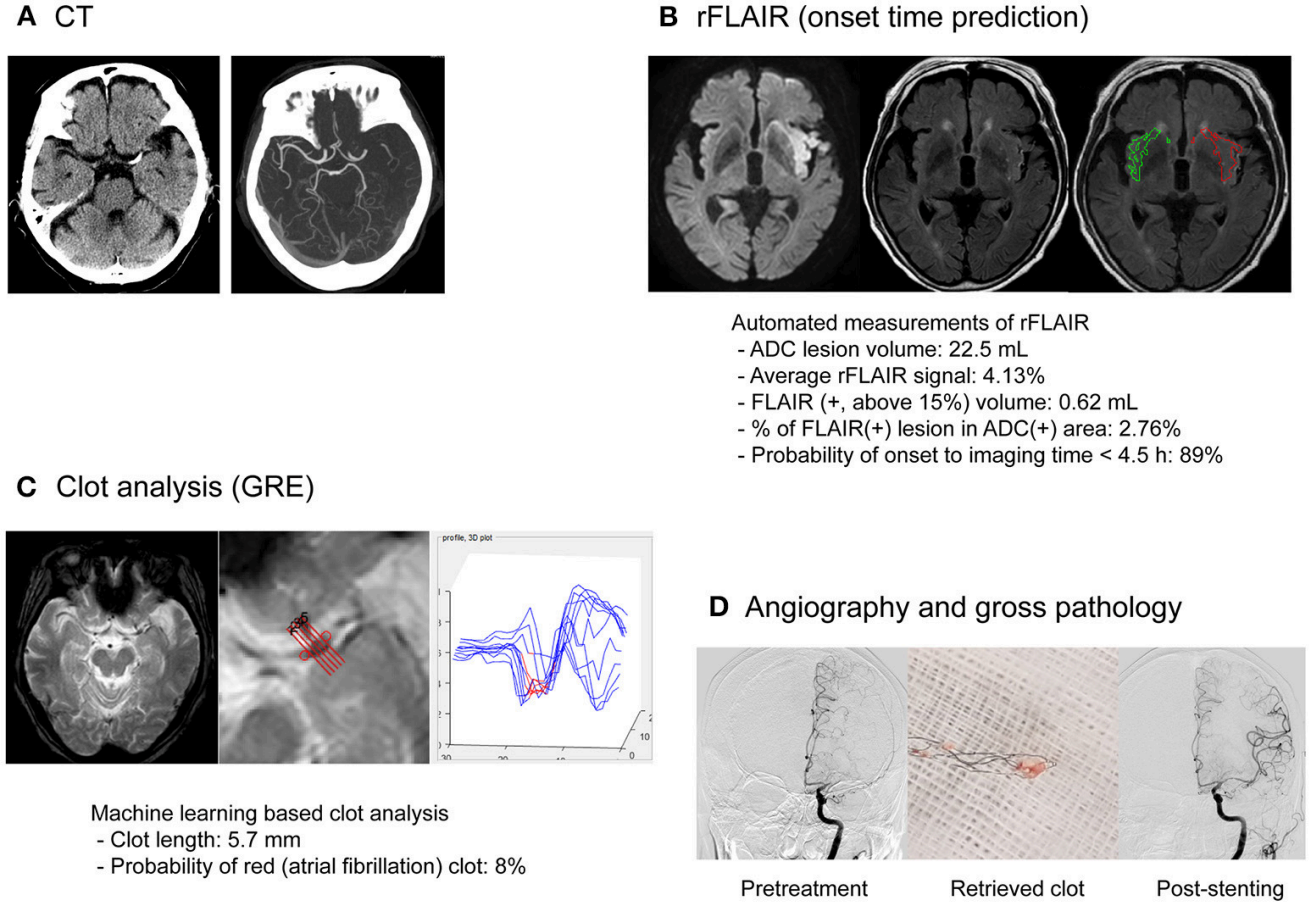
MRI-based endovascular treatment of a stroke patient with unclear symptom onset time and atherosclerotic occlusion of the middle cerebral artery. An 81-year-old male presented with right-sided weakness and global aphasia, with an unclear onset time. **(A)** On the CT image, left MCA occlusion was documented with an ASPECTS of 8. The patient underwent multimodal MRI using a fast (9 min) MRI protocol. **(B)** Minimal signal change was observed on FLAIR imaging and the probability of stroke onset within 4.5 h, based on relative FLAIR signal analysis, was high. **(C)** Clot analysis using GRE imaging and machine-learning techniques showed a low possibility of atrial fibrillation as cause of the clot. **(D)** Whitish clots were retrieved during endovascular treatment. Successful recanalization (mTICI grade 2b) was achieved with rescue stenting after repetitive reocclusion with a stentriever. His modified Rankin score at the 90th day was 1. CT, computed tomography; MCA, middle cerebral artery, ASPECTS, Alberta Stroke Program Early CT Score; ADC, apparent diffusion coefficient; FLAIR, fluid attenuation inversion recovery; GRE, gradient echo; mTICI, modified treatment in cerebral ischemia.

Aside from demonstrating a perfusion-diffusion mismatch and delineating penumbral and irreversibly infarcted regions, MRI could provide additional information on the blood–brain barrier (BBB), collaterals, and clot. MRI techniques for determining the age of the infarct was mentioned above. Representative cases are presented in Figures 2, 3.

#### Collateral flow

Better collaterals are associated with improved clinical and radiological outcomes, while poor collaterals are linked to hemorrhagic complications and poor recanalization rates after revascularization therapy for acute ischemic stroke (51). A collateral flow map derived from MRP source data can be generated by automatic post-processing (42). This study showed good correlation between MRI-based collateral grade and conventional angiography-based collateral grade, indicating that pretreatment MRI-based collateral evaluation could replace conventional angiographic evaluation in the angio-suite, which may require >20 min before EVT can be initiated. The role of MRI-based collateral imaging (FAST-COLL) is currently being tested in a prospective observational study (Clinicaltrials.identifier NCT02668627), to evaluate whether MRI-based collateral imaging is feasible and can predict the response to EVT in a wide range of patients with acute ischemic stroke. This study is evaluating the early infarct grow rate and eligibility according to the DAWN and DEFUSE 3 criteria, depending on the pretreatment MRI-based collateral grades. It is interesting that a significant proportion of patients who are eligible according to the DEFUSE 3 criteria did not meet the DAWN criteria for eligibility (20, 21).

#### Clot treatment

Successful reperfusion may be associated with the histopathology of occlusive thrombi, including the existence of atheromatous gruel and the proportion of erythrocyte components (52). Intracranial atherosclerosis is particularly prevalent in Asians, and is associated with frequent EVT failure. In this condition, adjuvant therapy, such as the use of a GP IIb/IIIa inhibitor or permanent stent placement, may be needed (53). Although previous studies have attempted to predict the response to revascularization therapy using CT imaging of the clot, a recent study showed a lack of association between CT-based clot images and the histopathology of thrombi, and stroke etiology (54). MRI can identify clots with high specificity and can measure the clot burden more accurately than CT images. Blooming artifacts caused by paramagnetic materials in GRE or susceptible weighted images have been associated with cardioembolic stroke (55, 56).

#### BBB derangement

GRE images are as accurate as CT at detecting acute hemorrhage in patients with acute stroke. BBB permeability dysfunction often precedes hemorrhagic transformation (57). Gadolinium contrast agents are routinely used to detect BBB disruptions in patients with strokes or tumors. Using a simple post-processing algorithm that employs pretreatment MRP source data, MRI permeability images can visualize BBB dysfunction and identify patients at risk of hemorrhagic transformation, with high specificity (57). A multicenter study tested various MRP-derived permeability measures in acute stroke patients and showed that MRI permeability images may be used in clinical practice (58). The multicenter DEFUSE 2 and MR RESCUE trials showed that the amount of BBB disruption seen on pretreatment MRI is associated with the severity of intracranial bleeding after EVT (59, 60).

### Machine learning

Machine learning is an approach used to achieve artificial intelligence goals. The field of artificial intelligence has evolved significantly with the introduction of a number of sophisticated algorithms, some of which are capable of self-learning. Application of artificial intelligence in the stroke field is increasing, and is used in the prediction of stroke [e.g., risk factors (61) and fine particulate matter (PM_2.5_) (62)] and pervasive health monitoring, by using smart monitoring devices embedded in the living environment (e.g., real-time monitoring via smartphone for adherence to oral anticoagulant treatment) (63). It can be particularly helpful in decision-making in every step of EVT for acute ischemic stroke: in clinical and imaging recognition of acute ischemic stroke in the ambulance or emergency room (64), and in predicting the outcome after EVT (65).

Machine learning-based assessment has advantages over simple visual estimation as follows. First, the application of deep learning to create an algorithm for automated detection of abnormal neuroimaging findings can improve inter-rater correlation. For example, the NCCT ASPECTS is widely used worldwide and there have been efforts to increase the inter-rater reliability, including implementation of a teaching program. However, inter-rater reliability has been reported to be low, particularly in a hyperacute setting. One recent systematic review showed that, in patients considered for EVT, there may be insufficient agreement between clinicians for the ASPECTS to be used reliably as a criterion for treatment decisions (66). Although the agreement could be higher among stroke experts than stroke trainees, it may still be lower than that achieved with a machine learning-based decision algorithm developed using various neuroimaging data. A computer algorithm that would automatically read a brain CT scan and automatically generate an ASPECTS (e-ASPECTS) may improve reliability in terms of calculating the score. A multicenter trial has shown that e-ASPECTS was non-inferior to neuroradiologists in determining the ASPECTS score using NCCT images obtained from acute stroke patients (67).

Second, machine-learning techniques facilitate the merging of information from various MR sequences. Integration of information from several MRI sequences could improve the role of MRI-based triage for EVT. For example, although combining DWI and FLAIR data showed high predictive values for identifying patients within 4.5 h of symptom onset, adding information on collaterals or perfusion improved the accuracy of predicting the time from symptom onset within 4.5 h (68, 69). Combining data from quantitative image analyses with other types of data (e.g., clinical or laboratory) can provide models that are helpful in the choice of work up, prediction of outcome or response to treatment.

Finally, because deep learning uses numerous imaging features that are most predictive for certain types of stroke pathophysiology, rather than explicitly detecting clinical features with which stroke physicians are familiar (e.g., conventional DWI), both physicians and patients have to trust a “black box” to determine a disease state. With increased numbers of MR sequences, many variables may influence how a machine defines referable stroke pathophysiologies. These include heterogeneous populations comprising different races (with different stroke subtypes, along with normal variations), heterogeneous prestroke conditions (e.g., preexisting atherosclerotic changes, white matter changes and other age-related changes) and novel features of specific sequences. Because the software does not detect the implication of the algorithm generated by the machine learning process, the stroke neurologist and radiologist need to provide the background and interpretation.

## Conclusions and perspectives

CT has been the standard neuroimaging modality for determining whether EVT should be applied due to the limited availability and standardization and time required for acquisition or post-processing of MRI data. However, more information may be available within the permitted time-windows if rapid acquisition of MRI data, automated fast post-processing and machine learning-based decision algorithms can be implemented. The role of imaging in EVT may shift from “go/no go” to “how to go.” With the advances in transformative technologies (such as machine learning and artificial intelligence) along with a better understanding of stroke pathophysiology and MRI physics, it is highly likely that multimodal MRI information could guide treatment strategies for patients with acute ischemic stroke.

As technology becomes more complex, selection of the technology used becomes more important. Stroke physicians will need to understand the evolution of such technology. Appropriate RCTs are required to verify the usefulness of imaging-based algorithms for the selection of EVT before these techniques can be incorporated into routine clinical practice.

## Author contributions

OB, the corresponding author, established the study idea, wrote the manuscript, and made critical revisions to the manuscript with substantive intellectual content. J-WC, JS, and Y-CK established the study idea, analyzed the data, and wrote the manuscript. W-SR, D-EK, W-KS, and G-MK established the database and made critical revisions to the manuscript with substantive intellectual content.

### Conflict of interest statement

The authors declare that the research was conducted in the absence of any commercial or financial relationships that could be construed as a potential conflict of interest.

## Abbreviations

ASIR: acute stroke imaging research
ASPECTS: Alberta Stroke Program Early CT score
DAWN: Diffusion-Weighted Imaging or Computerized Tomography Perfusion Assessment with Clinical Mismatch in the Triage of Wake-Up and Late Presenting Strokes Undergoing Neurointervention with Trevo
DEFUSE: Diffusion and Perfusion Imaging Evaluation for Understanding Stroke Evolution study
DWI: diffusion-weighted image
ECASS: European Cooperative Acute Stroke Study
EPITHET: Echoplanar Imaging Thrombolytic Evaluation Trial
ESCAPE: Endovascular treatment for Small Core and Anterior circulation Proximal occlusion with Emphasis on minimizing CT to recanalization times
EXTEND-IA: Extending the Time for thrombolysis in Emergency Neurological Deficits–;Intra-Arterial
FLAIR: fluid attenuation inversion recovery
HERMES: Highly Effective Reperfusion evaluated in Multiple Endovascular Stroke
IMS-III: Interventional Management of Stroke III
MR CLEAN: Multicenter Randomized Clinical trial of Endothelial treatment for Acute ischemic stroke
MRP: magnetic resonance perfusion
MR RESCUE: Mechanical Retrieval and Recanalization of Stroke Clots Using Embolectomy
REVASCAT: Randomized Trial of Revascularization With Solitaire FR Device vs. Best Medical Therapy in the Treatment of Acute Stroke Due to Anterior Circulation Large-Vessel Occlusion Presenting Within 8 h of Symptom Onset
SWIFT PRIME: Solitaire With the Intention For Thrombectomy as Primary Endovascular treatment for acute ischemic stroke
SYNTHESIS: intra-arterial vs. systemic thrombolysis for acute ischemic stroke.
